# Efficacy of the addition of interferential current to Pilates method in patients with low back pain: a protocol of a randomized controlled trial

**DOI:** 10.1186/1471-2474-15-420

**Published:** 2014-12-10

**Authors:** Yuri Rafael dos Santos Franco, Richard Eloin Liebano, Katherinne Ferro Moura, Naiane Teixeira Bastos de Oliveira, Gisela Cristiane Miyamoto, Matheus Oliveira Santos, Cristina Maria Nunes Cabral

**Affiliations:** Masters and Doctoral Programs in Physical Therapy, Rua Cesário Galeno 475, Tatuapé, CEP: 03071-000 São Paulo, SP Brazil

**Keywords:** Pilates-based exercises, Low back pain, Disability evaluation, Interferential current electrotherapy

## Abstract

**Background:**

Chronic low back pain is one of the four most common diseases in the world with great socioeconomic impact. Supervised exercise therapy is one of the treatments suggested for this condition; however, the recommendation on the best type of exercise is still unclear. The Pilates method of exercise is effective in reducing pain and disability in these patients, as well as the analgesia promoted by interferential current. Currently, the literature lacks information on the efficacy of the association of these two techniques in the short- and medium-term than performing one of the techniques isolated. The objective of this study will be to evaluate the efficacy of adding interferential current to the Pilates method exercises for the treatment of patients with chronic nonspecific low back pain in the short- and medium-term.

**Methods/Design:**

This study will be a randomized controlled trial with two arms and blinded evaluator, conducted at an outpatient Physical Therapy Department in Brazil. Patients with nonspecific chronic low back pain and pain equal to or greater than 3 in the Pain Numerical Rating Scale (0/10) will be randomly assigned to one of two groups: Group with active interferential current + Pilates (n = 74) will be submitted to the active interferential current associated to the modified Pilates exercises, and Group with sham interferential current + Pilates (n = 74) will be submitted to the sham interferential current associated with the modified Pilates exercises during 18 sessions. The outcomes pain intensity, pressure pain threshold, general and specific disability, global perceived effect and kinesiophobia will be evaluated by a blinded assessor at baseline, six weeks and six months after randomization.

**Discussion:**

Because of the study design, blinding of the participants and the therapists involved in the study will not be possible. The results of this study could contribute to the process of clinical decision- making for the improvement of pain and disability in participants with nonspecific chronic low back pain.

**Trial registration:**

ClinicalTrials.gov NCT01919268

**Electronic supplementary material:**

The online version of this article (doi:10.1186/1471-2474-15-420) contains supplementary material, which is available to authorized users.

## Background

Nonspecific low back pain is characterized by a mechanical pain of musculoskeletal origin, which lasts more than 12 weeks with no defined cause [1, 2]. The prognosis is considered moderately favorable, since 41% of the patients will be recovered by the end of the 12 months [3]. It is known that the shorter the period of chronicity of the low back pain, the greater the patient’s improvement regarding pain and disability [4]. In addition to the global prevalence estimated in 31% [5], low back pain is one of the four most common diseases around the world and is the leading cause of years lived with disability in developed countries [6]. In the United States, it is estimated that the direct and indirect costs [7] for treating this condition is about 84.1 to 624.8 billion dollars; therefore, it is a disease of great socioeconomic impact and the search for the most effective treatment is essential.

Supervised exercise therapy has been recommended by clinical practice guidelines as an effective intervention for the treatment of people with chronic low back pain [1, 8]. However, the most effective type of exercise is still unclear. The Pilates method of exercise is among the array of exercises available for the physical therapy practice. There are several systematic reviews on the use of the Pilates method in the treatment of low back pain, however the results on the effects of this technique are still conflicting. A recent systematic review [9] aimed to provide an update on the effectiveness of the Pilates method in the treatment of patients with chronic low back pain, as several relevant clinical trials were published in the last months. The review concluded that Pilates method exercises show a statistically significant improvement in pain and disability in the short-term and a clinically significant improvement in pain compared to usual care and physical activities. However, the Pilates method showed an equal effect compared to other forms of exercise and massage therapy, although these results are still conflicting.

Another resource widely used in clinical practice, as an adjunct in the treatment of low back pain, is the interferential current [10–15]. The interferential current is an alternating electric current of medium frequency and with amplitude-modulated at a low frequency [16]. Currently, there is a lack of studies investigating the mechanisms of analgesic action of interferential current, but the theory most often cited to explain the modulation of pain by interferential current is the gate control theory [17]. This theory suggests that stimulation of afferent fibers of large diameter (Aβ) promotes the activation of local inhibitory circuits of the dorsal horn of the spinal cord and thus, prevents the pain impulses carried by small diameter fibers (C and Aδ) to reach higher centers [18]. Studies have shown that analgesic electrical currents, whether of low or medium frequency, may reduce pain in patients with chronic low back pain in the short-term [12, 19–21]. However, current evidence on the efficacy of the association of interferential current therapy and supervised exercise, such as the Pilates method, are lacking.

Therefore, the primary objective of this study will be to evaluate whether the association of interferential current with Pilates exercises changes pain and disability in patients with nonspecific chronic low back pain in the short-term. The secondary objective will be to evaluate if the treatment changes specific disability, kinesiophobia and global perceived effect in the short- and medium-term, and pain and disability in the medium-term. The hypothesis is that the prior use of interferential current may improve exercise performance and collaborate with better clinical results, since patients with chronic low back pain have more difficulty performing the exercises due to pain and fear of a new, painful experience [22].

This will be a randomized controlled trial, with two arms and blinded evaluator, funded by São Paulo Research Foundation (FAPESP - 2013/17303-6), Brazil. The evaluations will be conducted at baseline, immediately after the intervention (active or placebo interferential current associated with Pilates exercises for six weeks) and six months after randomization.

## Methods/Design

### Participants, therapists and center

Participants with nonspecific chronic low back pain, aged between 18 to 80 years, of both sexes, and with pain intensity equal to or greater than 3 points, assessed by the Pain Numerical Rating Scale [23], will be recruited from the community through advertisements on websites, newspapers and TV shows. The inclusion criterion related to pain intensity was chosen, since participants with pain intensity inferior to 3 would not benefit from the clinical effect of the interferential current. Patients with any contraindications to perform exercises in accordance with the guidelines of the American College of Sports Medicine [24], with severe spinal diseases (such as fractures, tumors and inflammatory diseases), previous surgery on spine, nerve root compression, pregnancy, infection and/or skin lesions at the site of the application of the interferential current, with cancer, cardiac pacemaker, with changes in sensitivity or allergy in the region of electrode placement, under physical therapy treatment for chronic low back pain in the past six months and who are regularly involved with a Pilates program, will be excluded from the study. The eligibility criteria will remain unchanged after inclusion of the first participant in the study. In addition, the participants will be compensated for their transportation at the end of the study.

The participants will be taken care of by four physical therapists; two of them will be responsible for implementing the interferential current and assign participants to interventions (NTBO and MOS) and two responsible for the Pilates exercises (YRSF and KFM). The physical therapists responsible for implementing the Pilates exercises have specific training and a minimum of four years of experience with the method. In addition, these physical therapists will be blind with respect to which group the participant has been allocated. Participant assessment will be performed by another physical therapist that will not be involved in the treatment (GCM). The treatment sessions will be conducted at the outpatient Physical Therapy Clinic at Universidade da Cidade de São Paulo, in São Paulo, Brazil.

### Interventions

Participants will be randomly allocated in two intervention groups: 1) Group with active interferential current and Pilates exercises: this group will receive application of the active interferential current associated with the modified Pilates exercise; and 2) Group with sham interferential current and Pilates exercises: this group will receive the sham treatment of interferential current associated with the modified Pilates exercise. The treatment received by both groups will be carried out three times a week for six weeks consecutively. The sessions will be held individually. In the first two weeks, participants will be treated only with the active or sham interferential current to possibly reduce the pain and facilitate the performance of the exercises. In the next four weeks, immediately after the completion of electrotherapy sessions, the modified Pilates exercises will be added.

The interferential current will be delivered using a generator of alternating currents of medium frequency from the Brazilian Industry Medical Equipment (IBRAMED, São Paulo - SP, Brazil). Both groups will receive the bipolar mode, with two channels (4 self-adhesive electrodes, 50 × 90 mm) over the area of pain [25], with the following parameters: carrier frequency of 4 kHz; amplitude-modulated frequency (AMF) = 100 Hz; sweep frequency = 50 Hz; swing pattern 1:1 for 30 minutes [10]. In the Group with active interferential current and Pilates exercises, the physical therapist will be responsible for increasing the amplitude of the current until the participant reports feeling a “strong but comfortable tingling”. Every five minutes, the therapist will query the participant to ensure that the “strong but comfortable tingling” is maintained. In the case of decreased sensation, the amplitude of the current will be increased until the participant reaches the previous feeling [26]. In the Group with sham interferential current and Pilates exercises, all parameters will be adjusted as described, the audible alarm will be activated but the current amplitude will remain unchanged. The physical therapist responsible will ask if the participant is comfortable every five minutes, but without increasing the amplitude of the current.

After the first two weeks, participants in both groups will undergo a program of modified Pilates exercises that will be conducted on the floor and using the Cadillac, Reformer, Ladder Barrel and Step Chair equipment (Metalife, São José – SC, Brazil). In the first session of the Pilates exercises, participants will receive guidance through the method and will be trained to activate the powerhouse, that is to isometrically contract the transversus abdominis, multifidus and pelvic floor muscles associated with expiration. These muscle contractions will be performed throughout all exercises. The exercises have three levels of difficulty: basic, intermediary and advanced (Additional file 1). The level of difficulty will be set individually and the participants’ progress will be dependent on how well they perform 10 repetitions of the exercises without postural compensations [27, 28]. The strategy to prevent bias from the intervention is the individual monitoring of the participant by a trained physical therapist and the control of the level of exercise difficulty presented by the participant, since the treatment of chronic low back pain with the modified Pilates exercise has proven to be a safe intervention [29–31]. Moreover, the exercise program ends after the 6-week intervention, without any additional treatment, since both groups will receive the treatment according to the guidelines [1, 8] and the allocated interventions will not be modified. Participants that may need additional interventions will be referred to the outpatient Physical Therapy Clinic from the Universidade Cidade de São Paulo. During the study, the participants will be allowed to use their usual medication and this information will be monitored during the reassessments at six weeks and six months.

### Outcomes

The primary outcomes of the study are pain intensity, pressure pain threshold and disability at the 6-week follow-up after randomization. Secondary outcomes are global perceived effect, specific disability and kinesiophobia at the 6-week and 6-month follow-up after randomization, and pain intensity and disability at the 6-month follow-up after randomization.

Pain intensity will be evaluated using the Pain Numerical Rating Scale (0–10 points), with zero being “no pain” and 10 being “pain as bad as could be”. The participant must rate the average pain in the last seven days [32, 33].

Pressure pain threshold will be measured using the digital pressure algometer (Somedic Inc., Hörby, Sweden). Before data collection, the intra-rater reliability of the pressure pain threshold at the body landmarks used in the study were tested. The intra-rater reliability for the tibialis anterior muscle and the muscles of the lumbar region were 0.85 and 0.99, respectively, showing excellent intra-rater reliability. The algometry points will be marked using a measuring tape and pen. The participant will be seated in a chair without back support and the bony prominences over the vertebrae will be palpated bilaterally. The first landmark is located 5 cm laterally from the spinous processes of L3 [34] and the second landmark is 5 cm laterally from the spinous processes of L5 [35]. The third landmark is over the tibialis anterior muscle of the right leg in order to evaluate the segmental hyperalgesia [36, 37]. To measure the pressure pain threshold, the circular algometer probe will be positioned at an angle of 90° to the skin and pressed with a rate of about 50 kPa/s. The participant will be orientated to press and release a button when the sensation of pressure changes to a sharp pain. In this study, three repetitions of the measurement (in kPa) will be taken at each point, with an interval of 30 seconds between them [38]. The average of the three measurements will be used for analysis. The tests will be demonstrated two times using, as an example, the extensor muscles of the forearm of the dominant limb. If necessary, a third demonstration will be conducted. For participants without any pain at a pressure of 1000 kPa, this level of pressure pain threshold will be used for analysis.

The Roland Morris Disability questionnaire will be used to measure disability of the participant with regard to the physical limitations caused by pain in the lumbar spine in the last 24 hours. It is a 24-item questionnaire with each answer scaled either “yes” or “no” and is related to the activities of daily living of the participant - each affirmative response corresponds to one score. The final score is determined by the sum of all the scores obtained for each question. Scores close to zero are the best results (lower disability) and scores near 24 are the worst results (higher disability). Scores greater than 14 points are indicative of severe impairment of the back [32, 39–41].

The global perceived effect will be evaluated by the Global Perceived Effect scale that compares the onset of symptoms with the last few days. It is an 11-point numerical scale, ranging from -5 to 5, with -5 being “vastly worse”, 0 being “no change” and 5 meaning “completely recovered”. Higher scores indicate greater recovery from the condition [32, 42].

Specific disability will be assessed using the Patient-Specific Functional scale and the participants will be asked to identify three important activities that they feel incapable of or have difficulty performing due to chronic low back pain. The participants will indicate on an 11-point scale (0–10) how capable they feel performing specific activities, with 0 representing “unable to perform the activity” and 10 representing “able to perform the activity at preinjury level”. The average of the scores for the three activities will be calculated. The higher the score, the greater the functional ability of the participant [32, 43].

Kinesiophobia will be measured using the Tampa scale for Kinesiophobia that comprises 17 questions that address pain and symptom severity. The scores range from 1 to 4 points, where 1 represents “strongly disagree”, 2 represents “partially disagree”, 3 represents “partially agree”, and 4 represents “strongly agree”. For the overall final score, the scores for questions 4, 8, 12, and 16 must be inverted. The final score ranges from 17 to 68 points, with higher scores indicating a higher degree of kinesiophobia [44–46].

All outcome measures were previously adapted cross-culturally into Brazilian-Portuguese language, and the measurement properties of these instruments were tested with participants with low back pain in Brazil [32, 39, 40, 44, 45], and the results were acceptable and equivalent to those of the original versions in English.

### Procedures

Participants with nonspecific chronic low back pain will be recruited for the study. The participants will be assessed by a blinded evaluator, with prior training and experience in the field, for confirmation of eligibility criteria. In addition, demographic and anthropometric data will be obtained and other information such as the use of pain medications and the realization of any other treatment for low back pain. Eligible participants will be informed about the objectives and procedures of the study and following, the primary and secondary outcomes will be collected. Written informed consent will be obtained from all participants.

Randomization will be performed by a researcher not involved in the recruitment and treatment of the participants (CMNC), using the random number generation from Microsoft Excel for Windows. The allocation will be concealed by using consecutively numbered, sealed, opaque envelopes. After evaluation, eligible participants will be referred to the physical therapists responsible for the electrotherapy treatment that will randomly distribute the participants in the treatment groups.

The primary and secondary outcomes will be assessed at three different terms: baseline, 6-week follow-up and 6-month follow-up. In addition, at the last two assessments the participants will be instructed to refrain from providing treatment information to the evaluator. Only the pressure pain threshold obtained with the algometry will be performed at baseline and at the 6-week follow-up. This procedure aims at minimizing the loss of participants on returning visits at the 6-month follow-up for assessment, since the algometry requires the physical presence of the participant while the questionnaires and scales can be reapplied over the phone. Due to the nature of the intervention, it will not be possible to blind the participants and the therapists responsible for the implementation of the interferential current. Participant recruitment began in October 2013 and has a target date of February 2015 for completion. Table 1 shows the timeline of the study.

**Table 1:** **Timeline for the schedule of enrolment, interventions, and assessments**

Outcomes	Enrolment	Before randomization	Intervention period (6 weeks)	6-week follow-up after randomization	6-month follow-up after randomization
Eligibility criteria	X				
Demographic data	X				
Informed consent	X				
Primary outcomes					
Pain intensity		X		X	
PPT		X		X	
Disability		X		X	
Secondary outcomes					
Pain intensity					X
Disability					X
GPE		X		X	X
Specific disability		X		X	X
Kinesiophobia		X		X	X
Interventions					
Active IC + Pilates			X		
Sham IC + Pilates			X		

PPT: Pressure pain threshold; GPE: Global perceived effect; IC: Interferential current.

### Data analysis

A committee for data monitoring will consist of two authors from the study (CMNC and REL) that are not involved with data collection and have no conflict of interest. Auditing of the randomization process of the participants in the intervention groups will be held monthly. Statistical analysis will be performed by a researcher who will receive the data encoded (REL). All data will be double-entered prior to the analysis. The mean effects of the interventions and the group differences for all the outcomes will be calculated using linear mixed models. The linear mixed models include terms for the treatment groups, time, and interaction terms “treatment group” and “time”. The term “time” will be coded as three categorical variables: baseline, 6-week follow-up and 6-month follow-up. No interim analysis will be performed. The analysis will follow the intention-to-treat principles. If a participant abandons the treatment, no additional outcome will be collected and the missing data will not be replaced. For all statistical analyses, the level of confidence will be set at 5%. The IBM Statistics Package for the Social Science (SPSS) version 19 for Windows will be used in the analysis.

### Sample size calculation

This study is designed to detect a clinically important difference between groups of one point in the Pain Numerical Rating scale (estimate for standard deviation = 1.84 points), 100 kPa in the pressure pain threshold measured with the algometer (estimate for standard deviation = 110 kPa) and four points in disability assessed by the Roland-Morris Disability questionnaire (estimate for standard deviation = 4.9 points). The following specifications will be considered: α = 0.05, statistical power of 80% and follow-up loss of 15%. The sample size calculation resulted in 148 participants that will be randomly allocated into two intervention groups.

### Ethics

Participants that agree to participate in the study will sign two copies of the informed consent, one that will be kept in our records and one for the participant. The collected data will be stored in locked cabinets and only the blinded evaluator will have access to that information. Later, the data will be entered and saved on computers with password protection to ensure confidentiality. Eligibility criteria, outcomes and analysis will not be modified after the enrolment of the first participant.

This protocol was approved by the Research Ethics Committee from Universidade Cidade de São Paulo (CAAE 18034113.7.0000.0064) and was prospectively registered at the Clinical Trials Registry (NCT01919268).

The São Paulo Research Foundation (FAPESP) provided financial support for the purchase of the materials for conducting the study (2013/17303-6). The funder had no part in designing the study and in its implementation, analysis, data interpretation and presentation of the results.

## Discussion

The results of this study will enhance our knowledge on the efficacy of combining the interferential current with the modified Pilates exercises in participants with chronic low back pain. The existing literature on the use of Pilates exercises have shown significant effects in reducing pain and disability. However, these effects were only maintained in the short-term [47]. The same results were observed with interferential current, which apparently is more effective than placebo when combined with other therapies [48] in reducing pain. This effect was also only maintained in the short-term. Therefore, it is assumed that the combination of these two techniques could facilitate the participant’s ability to perform the exercises more efficiently, resulting in a significant clinical improvement that could be maintained in the medium-term. Thus, the results of this randomized controlled trial will help physical therapists in the process of clinical decision-making.

The present study will be conducted with a satisfactory sample size to detect a significant clinical effect of treatment with a low risk of bias, since it was designed to obtain a score of eight on the PEDro scale. Another feature of the study is its pragmatism since the Pilates exercises will be individualized and will respect the condition of the participants. The exercises will follow a handout created by researchers with approximately 130 exercises divided into levels of progression (Additional file 1), which increases the clinical relevance of the results.

Limitations of this study are related primarily to the inability to blind the therapists and participants of the study. The therapists applying the interferential current must be aware to the type of electrotherapy used. In addition, to minimize bias and therapist interference, the therapists conducting the Pilates exercise session will be blinded to the electrotherapy intervention. Since the participants are involved in the Pilates exercise, they may not be entirely blind. However, participants will be unaware of which group of electrotherapy they were allocated and will be asked to refrain from talking to each other and from talking about the resources used in the treatment. The evaluator and the therapists responsible for the Pilates method will be aware of the allocation only after data analysis.

We are currently at the stage of data collection. A total of 116 participants have been evaluated at baseline, 90 participants have been evaluated immediately after treatment (6-week follow-up after randomization) and 30 participants have been assessed at the 6-month follow-up after randomization. Up to this time, five losses were recorded at the 6-week follow-up and two losses in the 6-month follow-up. Publication of the results and release of the spreadsheet with the data encoded is expected to occur during the first semester of 2015.

## Electronic supplementary material

Additional file 1:Applied exercises divided according to the level of progression.(DOC 2 MB)
