# Supplementary material for: Efficacy of the addition of interferential current to Pilates method in patients with low back pain: a protocol of a randomized controlled trial
Source: BMC Musculoskelet Disord. 2014 Dec 10;15:420. doi: 10.1186/1471-2474-15-420 (PMC4295299; doi:10.1186/1471-2474-15-420)
Supplement: Supplementary file 1 — Additional file 1:Applied exercises divided according to the level of progression.(DOC 2 MB) [file 12891_2014_2358_MOESM1_ESM.doc]

**Additional file 1 – Applied exercises divided according to the level of progression.**

| **Exercises for the Back (12)** | | |
| --- | --- | --- |
| **Basic** | **Intermediary** | **Advanced** |
| *Bridge Variant* (pelvic mobilization)  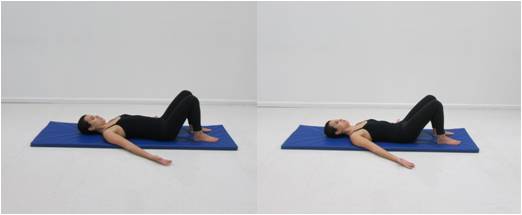  Objective: mobilize the pelvis and provide corporal awareness for the powerhouse contraction. | *Bridge Variant*  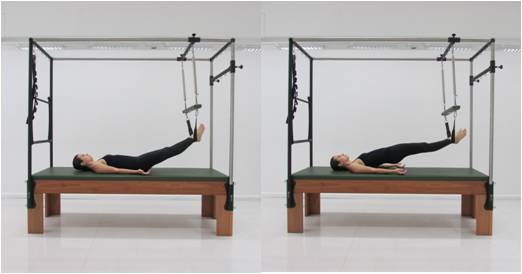  Objective: mobilize the pelvis and vertebral column and strengthen the hamstrings and gluteus muscles. | *Bridge Variant*  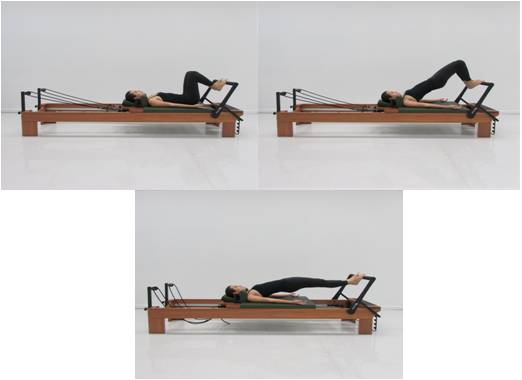Objective: strengthen the hamstrings, quadriceps femoral, calf, iliopsoas, sartorius, tensor fascia latae, pectineus and gluteus muscles. |
| *Bridge*  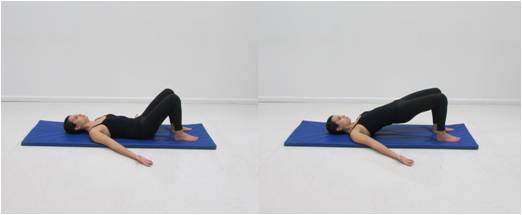  Objective: mobilize the pelvis and vertebral column and strengthen the gluteus and the hamstrings muscles. | *Bridge* *Variant*  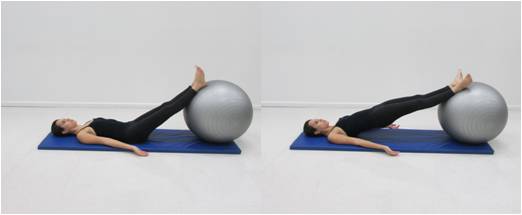  Objective: mobilize the pelvis and vertebral column and strengthen the gluteus, the hamstrings and the gastrocnemius muscles. | *Bridge* *Variant*  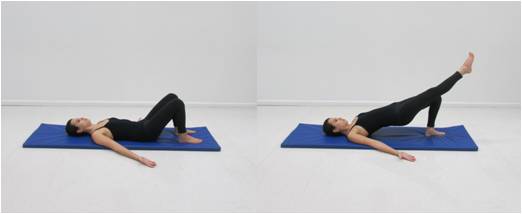  Objective: mobilize the pelvis and vertebral column and strengthen the hamstrings and the gluteus muscles. |
| *Rolling Back Down/Up* (heavy spring)  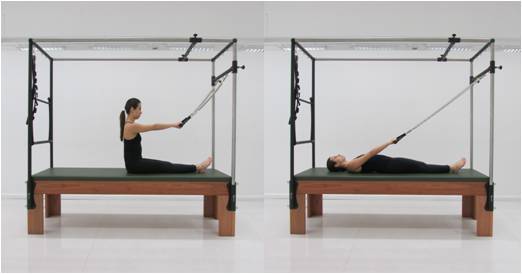  Objective: mobilize the vertebral column and contract the abdominal muscles associated with the powerhouse. | *Rolling Back Down/Up* (light spring)  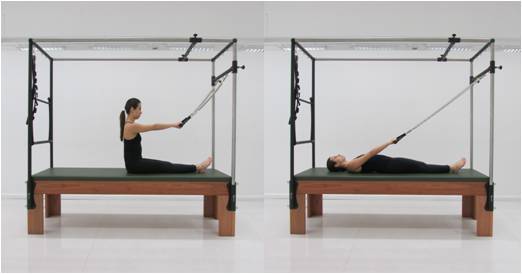  Objective: mobilize the vertebral column and contract the abdominal muscles associated with the powerhouse. | *Roll Up*  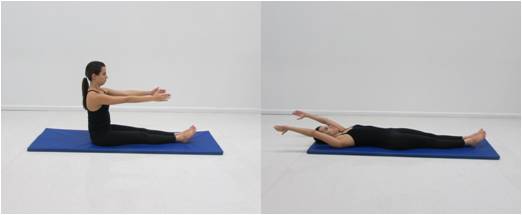  Objective: mobilize the vertebral column and strengthen the rectus abdominis and the obliquus externus muscles. |
| *Hamstring Stretch Variant*  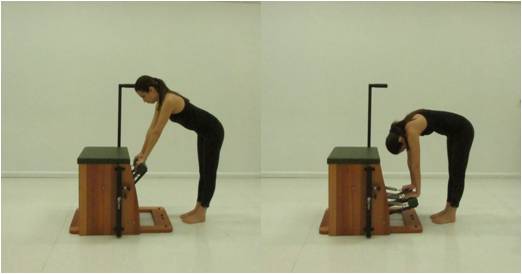  Objective: mobilize the vertebral column and lengthen the muscles from the posterior chain. | *Hamstring Stretch Variant*  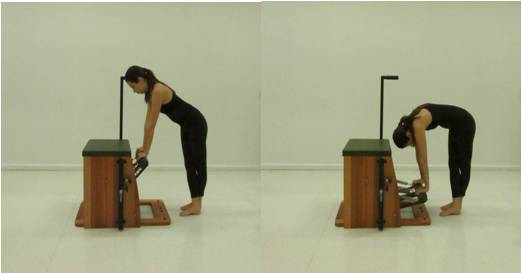  Objective: mobilize the vertebral column and lengthen the muscles from the posterior chain. | *Hamstring Stretch*  *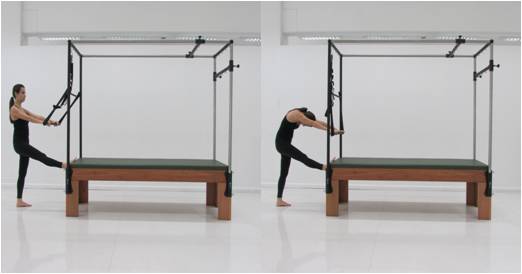*  Objective: mobilize the vertebral column and lengthen the muscles from the posterior chain. |
| *Stretches Front Variant – Barrel*  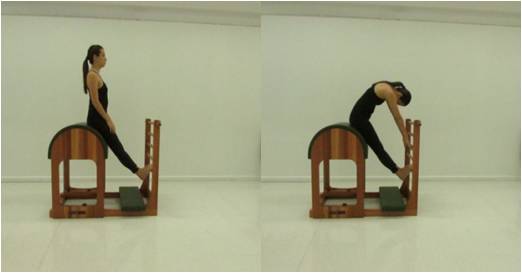  Objective: mobilize the vertebral column and lengthen the muscles from the posterior chain. | *Hamstring Stretch Variant*  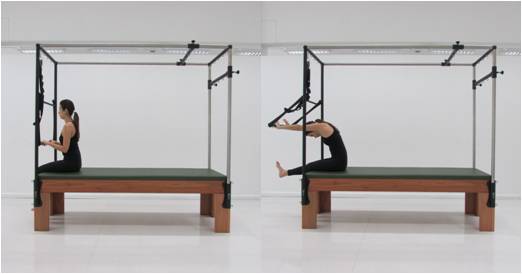  Objective: mobilize the vertebral column and lengthen the muscles from the posterior chain. | *Spine Stretch*  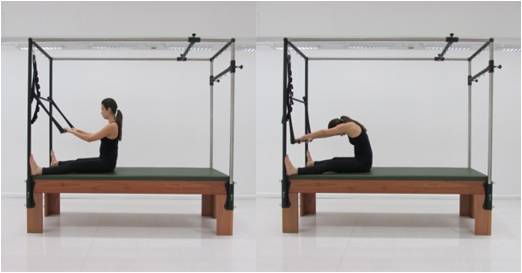  Objective: mobilize the vertebral column and lengthen the muscles from the posterior chain. |
| *The Cat*  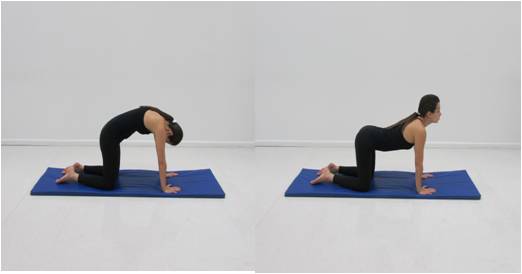  Objective: mobilize and provide coordination for the movements of the vertebral column. | *Knee Stretches Round*  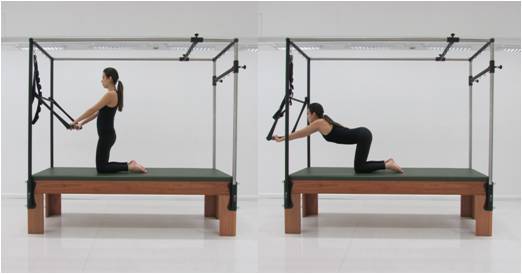  Objective: mobilize and provide coordination for the movements of the vertebral column. | *Spine Stretch*  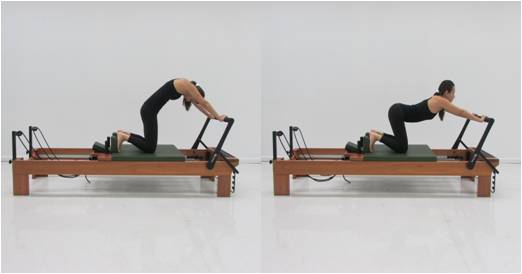  Objective: mobilize and provide coordination for the movements of the vertebral column. |
| *Swan Front*  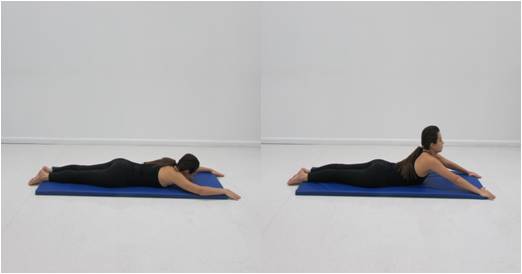  Objective: mobilize the vertebral column, lengthen the muscles from the anterior chain and strengthen the pectoralis major, triceps brachial and anterior deltoid muscles. | *Swan*  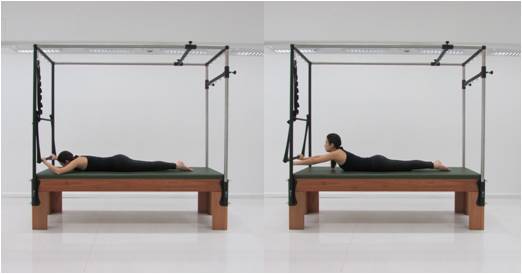  Objective: mobilize the vertebral column, control scapular elevation and strengthen the muscles from the posterior chain. | *Swan*  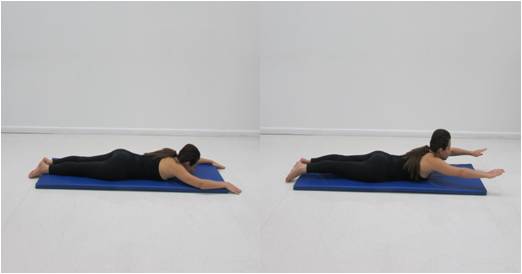  Objective: mobilize the vertebral column, control scapular elevation and strengthen the muscles from the posterior chain. |
| *Swan*  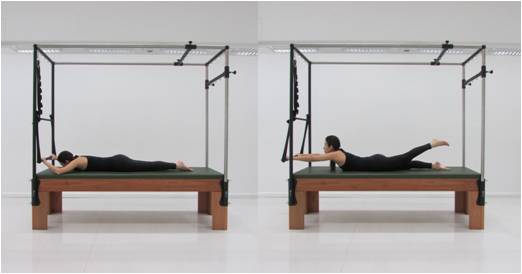  Objective: mobilize the vertebral column, control scapular elevation and strengthen the muscles from the posterior chain. | *Swan*  *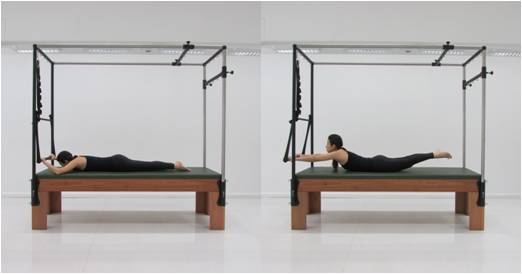*  Objective: mobilize the vertebral column, control scapular elevation and strengthen the paraspinal, deltoid, gluteus and hamstring muscles. | *Swimming*    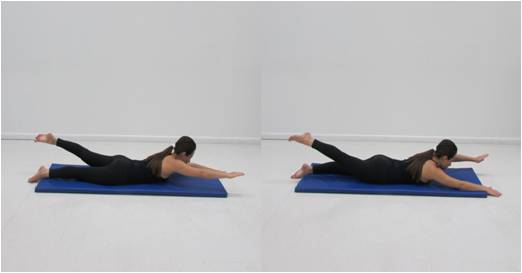  Objective: mobilize the vertebral column, control scapular elevation and strengthen the paraspinal, deltoid, gluteus and hamstring muscles. |
| *Swan Front* (trunk extension)  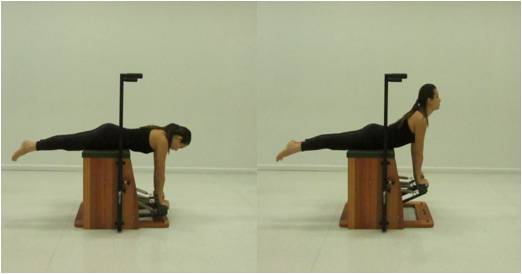  Objective: lengthen the muscles from the anterior chain and strengthen the paraspinal muscles. | *Swan Front* (unilateral without trunk extension)  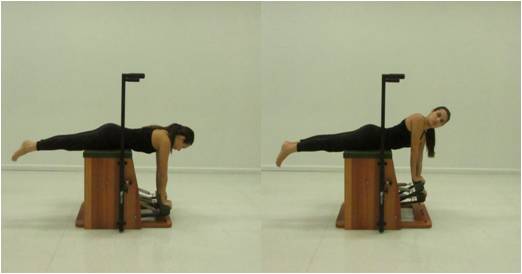  Objective: lengthen the muscles from the anterior chain and strengthen the spinal muscles. | *Swan Front* (unilateral with trunk extension)    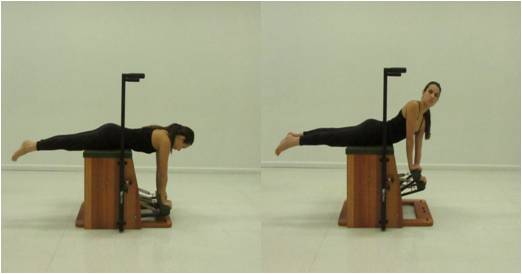  Objective: lengthen the muscles from the anterior chain and strengthen the paraspinal muscles. |
| *Tower*    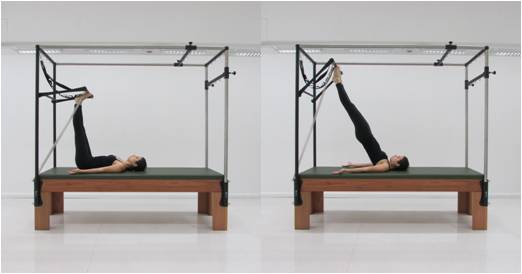  Objective: mobilize vertebral column, lengthen the muscles from the posterior chain and strengthen the abdominals, gluteus, hamstring and triceps surae muscles. | *Long Spine*    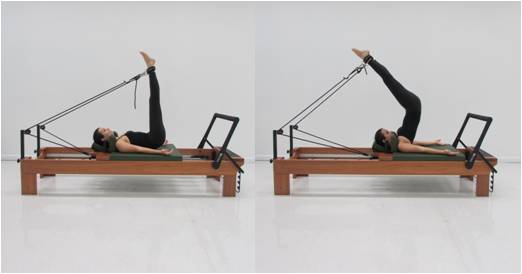  Objective: mobilize vertebral column, lengthen the muscles from the posterior chain and strengthen the rectus abdominis, rectus femoris, iliopsoas and gluteus muscles. | *Jackknife*  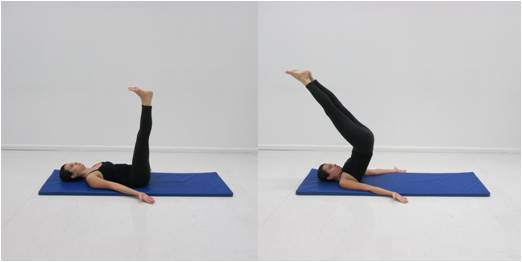  Objective: mobilize vertebral column and strengthen the abdominal, rectus femoris and iliopsoas muscles. |
| *Mermaid*  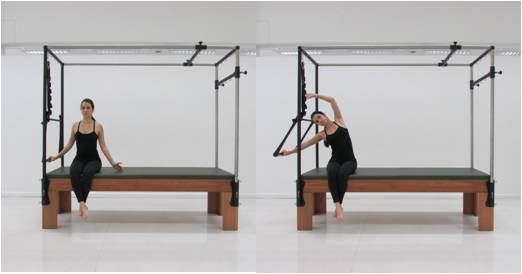  Objective: lengthen the muscles from the lateral chain and control scapular elevation. | *Mermaid*  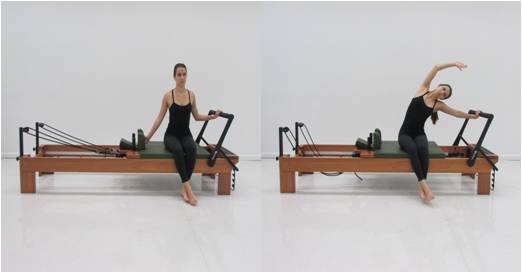  Objective: lengthen the muscles from the lateral chain and control scapular elevation. | *Side Arm Sit Variant*  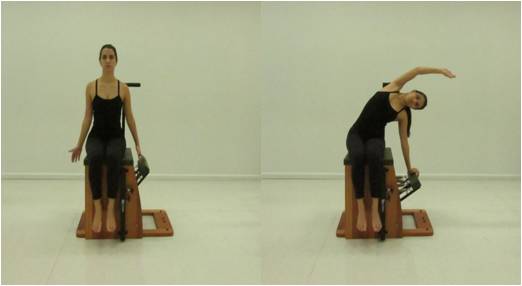  Objective: lengthen the muscles from the lateral chain and control scapular elevation. |
| *Rolling Back Variant*  *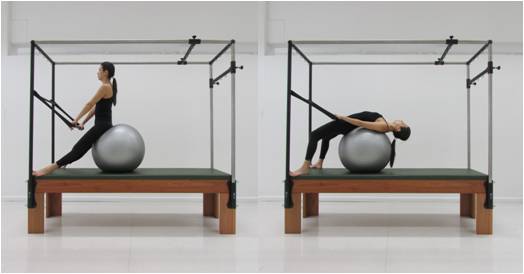*  Objective: mobilize the vertebral column and contract the abdominal muscles associated with the powerhouse. | *Rolling Back*  *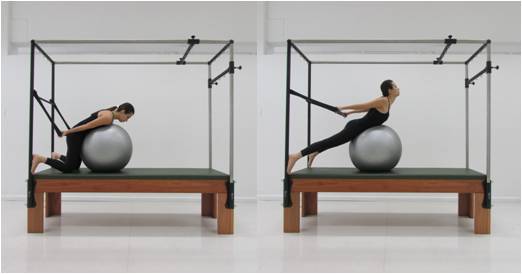*  Objective: mobilize the vertebral column, lengthen the muscles from the anterior-internal chain of the shoulder and contract the abdominal muscles associated with the powerhouse. | *Swan Variant*  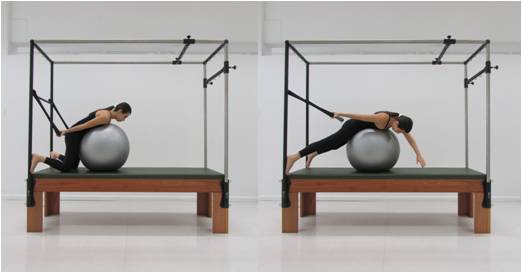  Objective: mobilize the vertebral column, control scapular elevation and lengthen the muscles from the anterior-internal chain of the shoulder. |

| **Exercises for the Lower Limbs (19)** | | |
| --- | --- | --- |
| **Basic** | **Intermediary** | **Advanced** |
| *Tower*  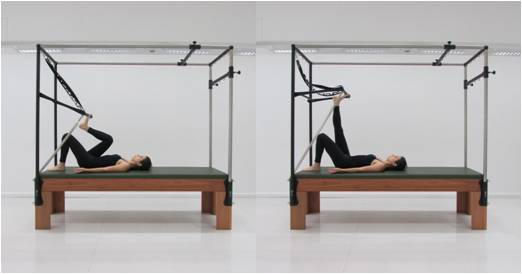  Objective: lengthen the muscles from the posterior chain and strengthen the quadriceps femoris and gluteus maximus muscles. | *Tower*    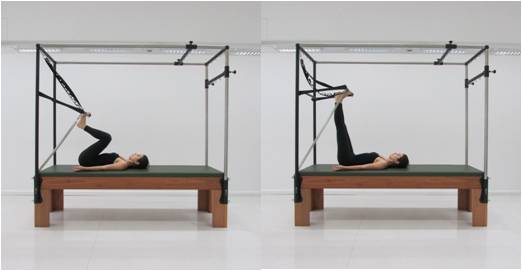  Objective: lengthen the muscles from the posterior chain and strengthen the quadriceps femoris and gluteus maximus muscles. | *Tower*    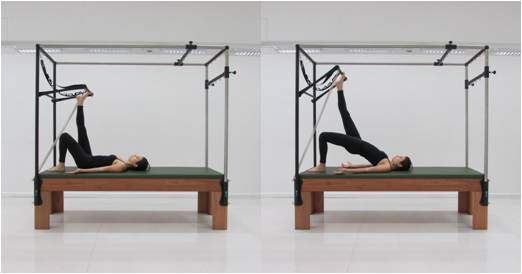  Objective: mobilize the vertebral column, lengthen the muscles from the posterior chain and strengthen the quadriceps femoris, hamstring and gluteus maximus muscles. |
| *Running*  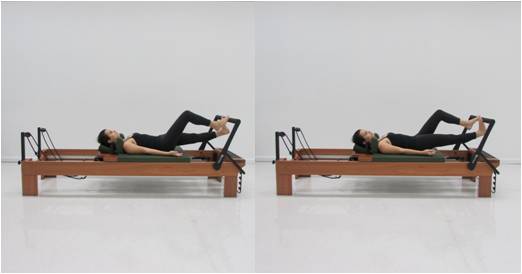  Objective: lengthen the muscles from the posterior chain and strengthen the quadriceps femoris, gluteus maximus and triceps surae muscles. | *Tower*    *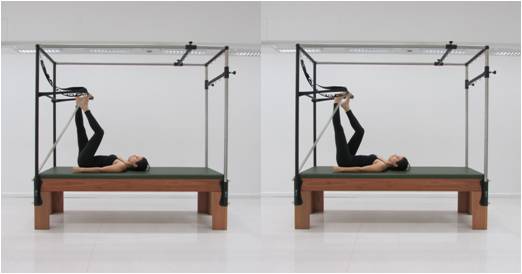*  Objective: lengthen the muscles from the posterior chain and strengthen the quadriceps femoris, gluteus maximus and triceps surae muscles. | *Stomach Massage Series*    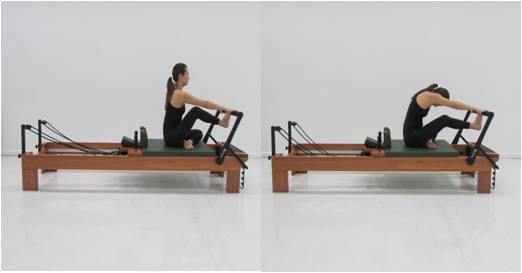  Objective: mobilize the vertebral column, lengthen the muscles from the posterior chain and strengthen the quadriceps femoris muscle. |
| *Footwork*  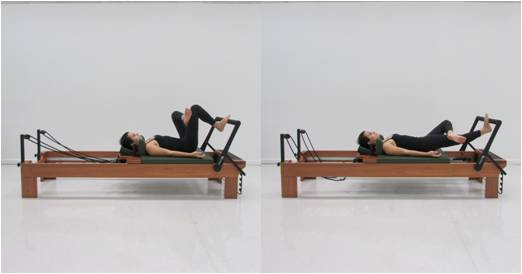  Objective: lengthen the gluteus maximus and piriformis muscles and strengthen the quadriceps femoris muscle. | *One Leg Up and Down*    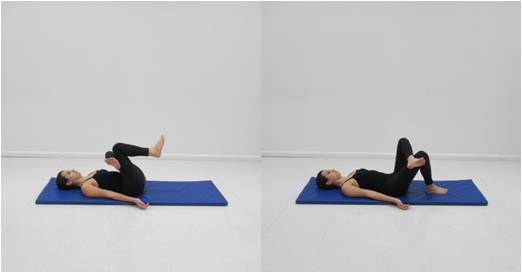  Objective: lengthen the gluteus maximus and piriformis muscles and strengthen the abdominal, iliopsoas and rectus femoris muscles. | *Tower*  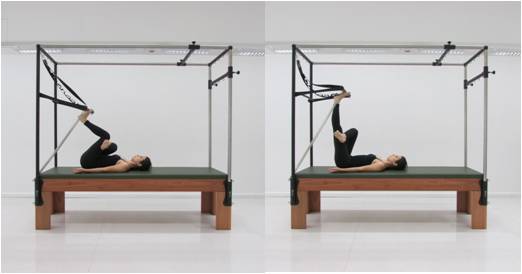  Objective: lengthen the gluteus maximus and piriformis muscles and strengthen the quadriceps femoris muscle. |
| *Footwork*  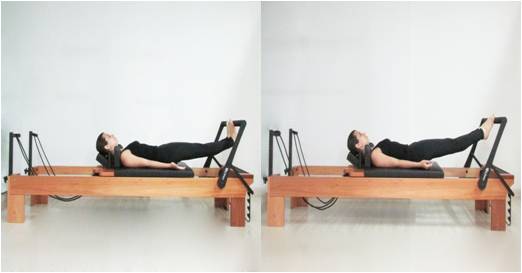  Objective: lengthen the hamstring and gastrocnemius muscles and strengthen the gastrocnemius muscle. | *Achilles Stretch*    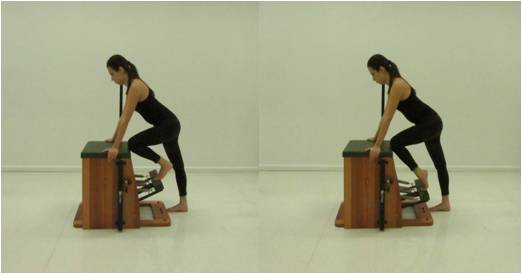  Objective: lengthen and strengthen the soleus muscle. | *Tower*  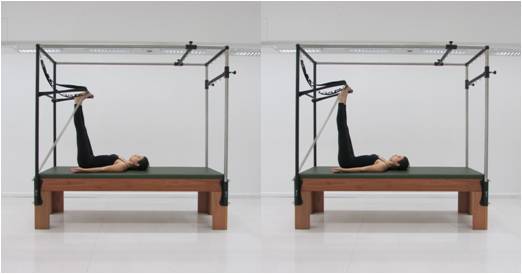  Objective: lengthen the hamstring and gastrocnemius muscles and strengthen the gastrocnemius muscle. |
| *Leg Circles*  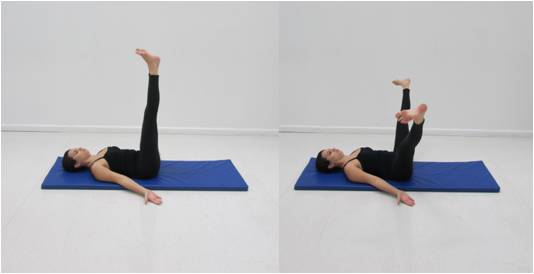  Objective: strengthen the quadriceps femoris, iliopsoas, sartorius, tensor fascia latae, pectineus, gluteus medius, gluteus minimus, adductor magnus and adductor longus and gracilis muscles. | *Leg Series Supine Circle*  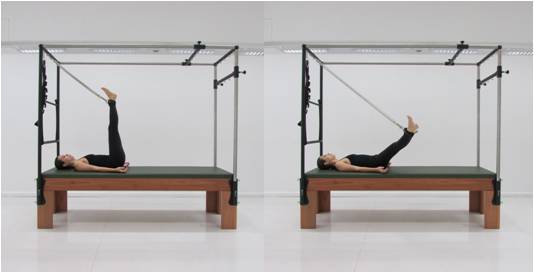  Objective: strengthen the gluteus maximus, hamstrings, adductor magnus and longus, pectineus and gracilis muscles. | *Leg Circles*    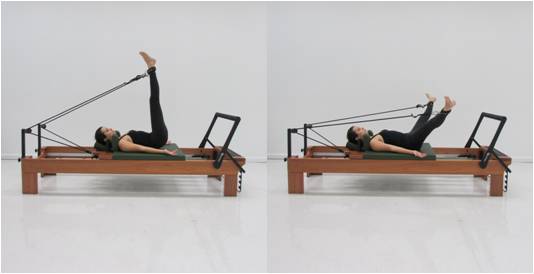  Objective: strengthen the gluteus maximus, hamstrings, adductor magnus and longus, pectineus and gracilis muscles. |
| *Leg Series Supine Lowers*  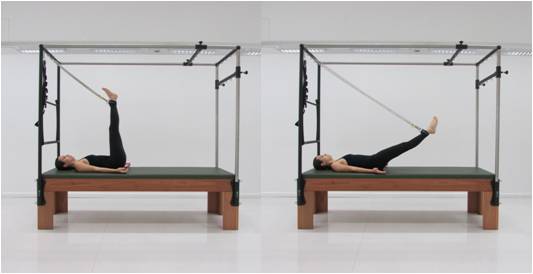  Objective: strengthen the gluteus maximus and hamstring muscles. | *Leg Lowers*  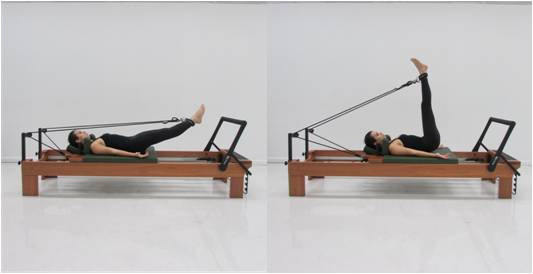  Objective: strengthen the gluteus maximus and hamstring muscles. | *Leg Series Supine Scissors*  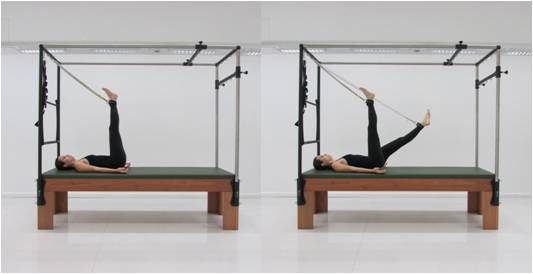  Objective: strengthen the gluteus maximus, hamstring, sartorius, gracilis and gastrocnemius muscles. |
| *Footwork Hells V Position*  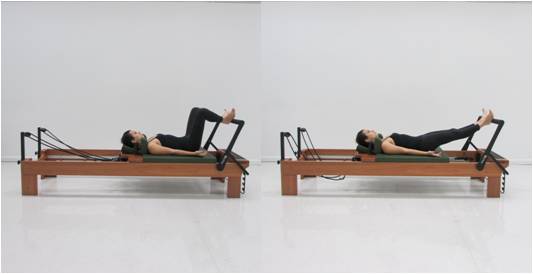  Objective: strengthen the quadriceps femoris, adductor magnus, adductor longus, pectineus and gracilis muscles. | *Leg Series Supine Frog*  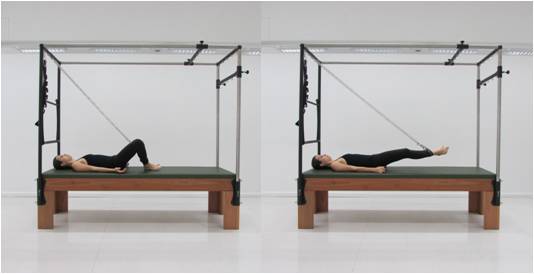  Objective: strengthen the quadriceps femoris, adductor magnus, adductor longus, pectineus and gracilis muscles. | *Knee Extension V Position*  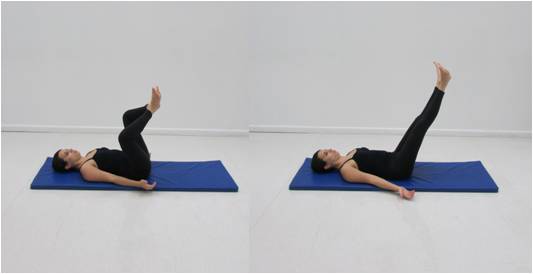  Objective: strengthen the quadriceps femoris, adductor magnus, adductor longus, pectineus, pes anserinus, rectus abdominis and obliquus externus muscles. |
| *Footwork Double Leg Pumps*    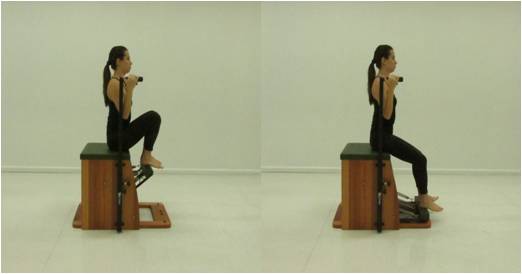  Objective: strengthen the quadriceps femoris and triceps surae muscles. | *Pump One Leg Front*    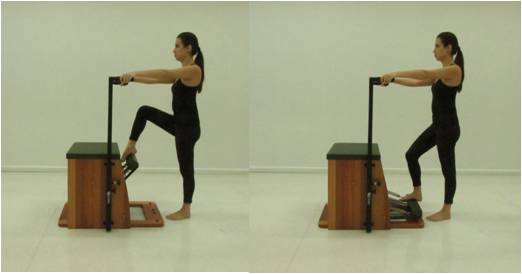  Objective: strengthen the quadriceps femoris and triceps surae muscles. | *Pumping One Leg*  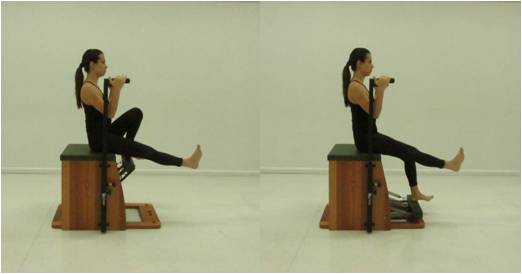  Objective: strengthen the quadriceps femoris, triceps surae, iliopsoas and sartorius muscles. |
| *Pump One Leg Side*  *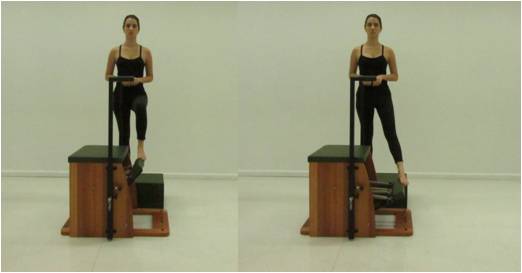*  Objective: strengthen the gluteus medius and tensor fascia latae muscles. | *Side Kicks Up and Down*    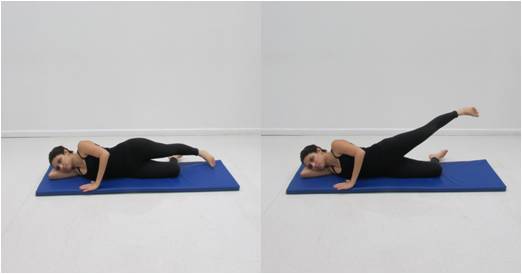  Objective: strengthen the gluteus medius and minimus, tensor fascia latae and sartorius muscles. | *Side Splits* (heavy spring)  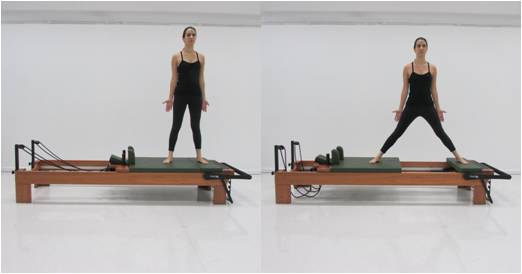  Objective: strengthen the gluteus medius and minimus, tensor fascia latae and sartorius muscles. |
| *Pump One Leg Side*    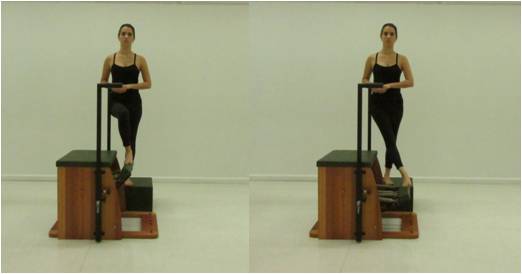  Objective: strengthen the gluteus maximus, hamstrings, adductor magnus, adductor longus, pectineus and gracilis muscles. | *Side Kicks Inner-Thigh* *Lifts*  *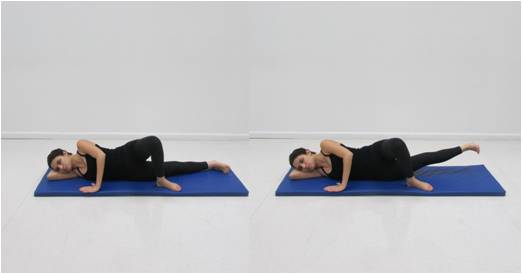*  Objective: strengthen the adductor magnus, adductor longus, pectineus and gracilis muscles. | *Side Splits* (light spring)    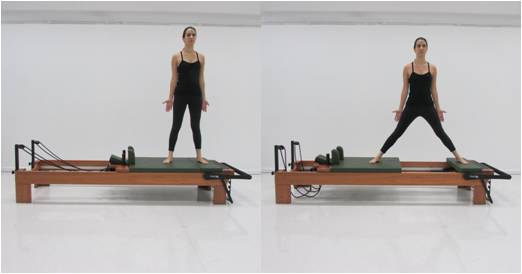  Objective: strengthen the adductor magnus, adductor longus, pectineus and gracilis muscles. |
| *Leg Series Supine*  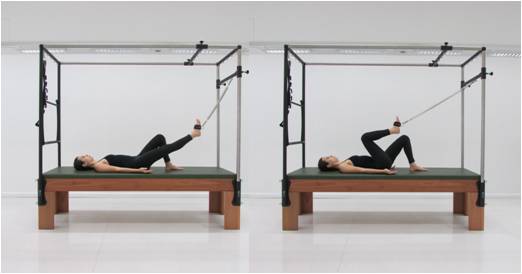  Objective: strengthen the iliopsoas, rectus femoris and rectus abdominis muscles. | *One Leg Up-Down*  *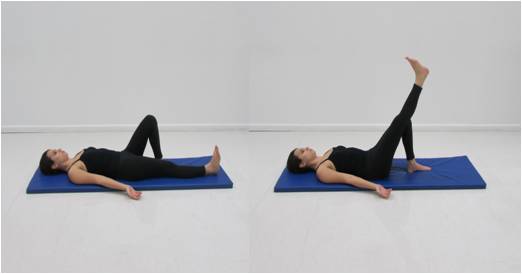*  Objective: strengthen the rectus femoris, iliopsoas, sartorius, tensor fascia latae and pectineus muscles. | *Leg Series*  *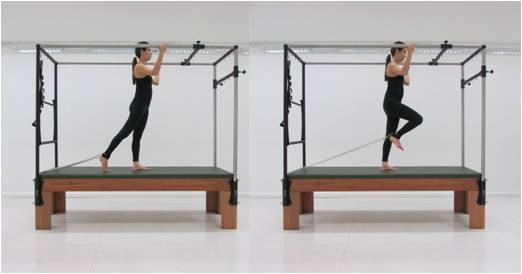*  Objective: strengthen the abdominal, quadriceps femoris and iliopsoas muscles. |
| *Footwork Toes*  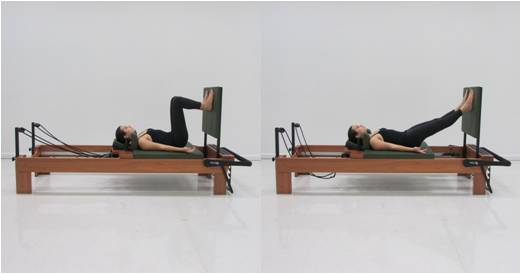  Objective: strengthen the quadriceps femoris muscle. | *Leg Series*  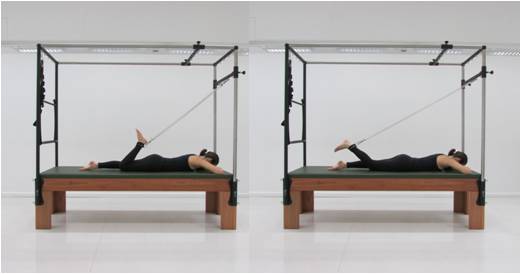  Objective: strengthen the quadriceps femoris muscle. | *Front Splints*    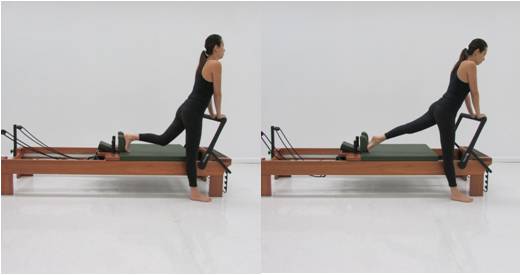  Objective: strengthen the quadriceps femoris and gluteus muscles. |
| *Footwork*  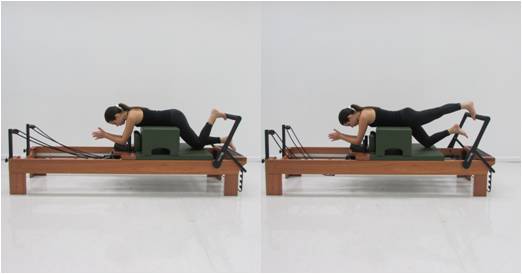  Objective: strengthen the quadriceps femoris and gluteus muscles. | *Fire Hydrant*  *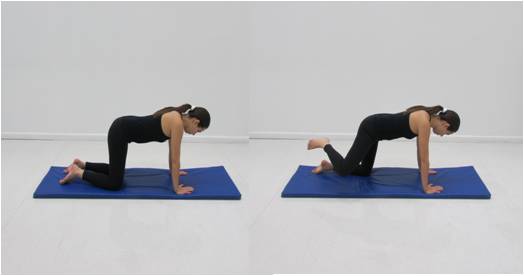*  Objective: strengthen the gluteus, piriformis, gemellae superior and inferior and obturator internus and externus muscles. | *Leg Series*  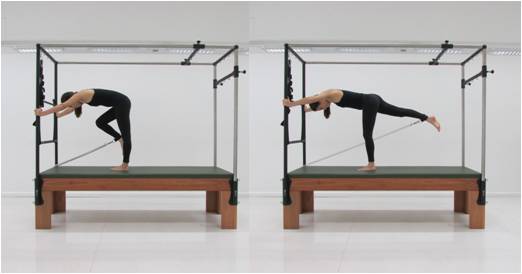  Objective: strengthen the gluteus maximus and hamstring muscles. |
| *Footwork*  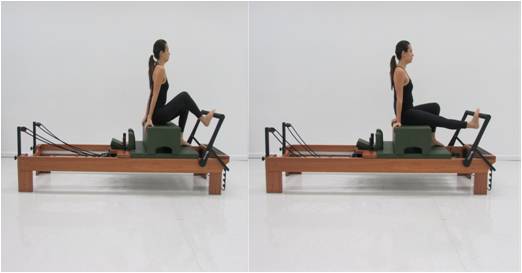  Objective: strengthen the quadriceps femoris muscle. | *Footwork*    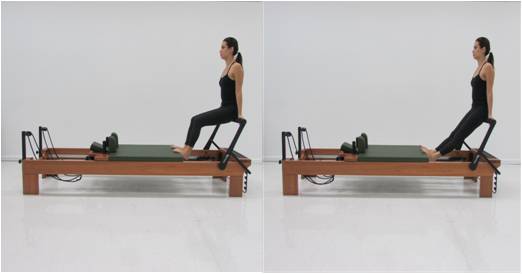  Objective: strengthen the quadriceps femoris muscle. | *Going Up-Front*    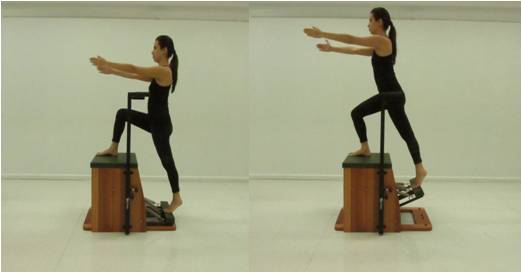  Objective: strengthen the quadriceps femoris and gluteus muscles. |
| *Leg Series*  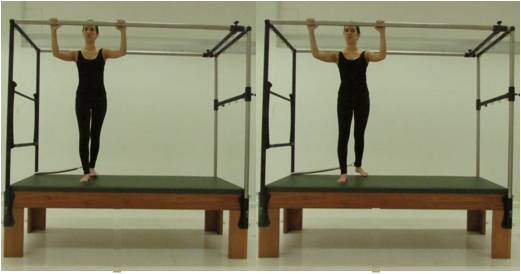  Objective: strengthen the gluteus medius and minimus, tensor fascia latae and sartorius muscles. | *Leg Series Diagonal*    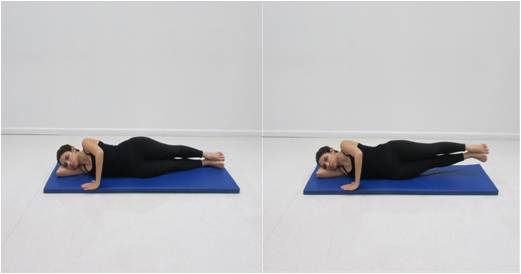  Objective: strengthen the gluteus medius and minimus, tensor fascia latae, adductor magnus, adductor longus and obliquus internus and externus muscles. | *Leg Series Diagonal*    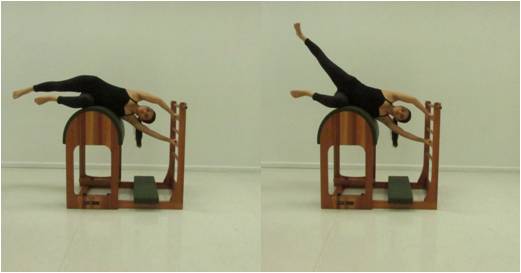  Objective: strengthen the gluteus medius and minimus, tensor fascia latae and sartorius muscles. |
| *Leg Series*  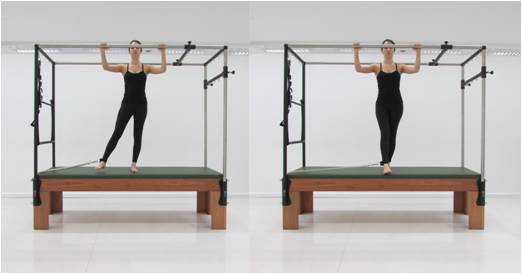  Objective: strengthen the adductor magnus, adductor longus, adductor minimus, pectineus and gracilis muscles. | *Leg Series On Side Up and Down*  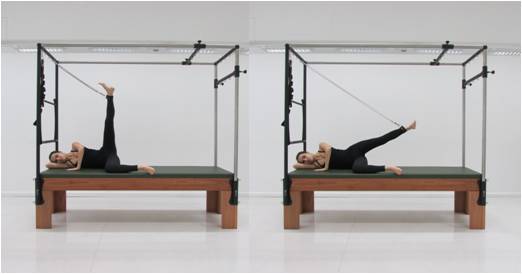  Objective: strengthen the adductor magnus, adductor longus, adductor minimus, pectineus and gracilis muscles. | *Scissor Leg Side Lying*  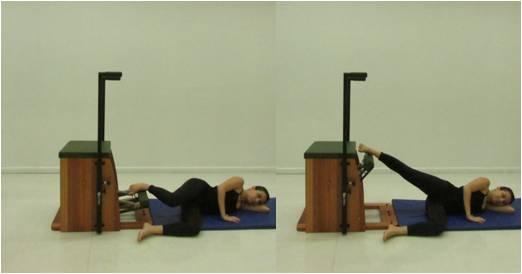  Objective: strengthen the adductor magnus, adductor longus, adductor minimus, pectineus and gracilis muscles. |
| *Teaser*  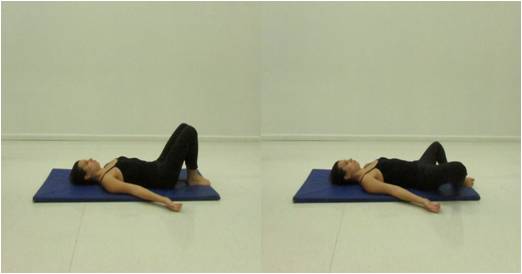  Objective: lengthen and strengthen the adductor magnus, adductor longus, adductor minimus, pectineus and gracilis muscles. | *Horse*    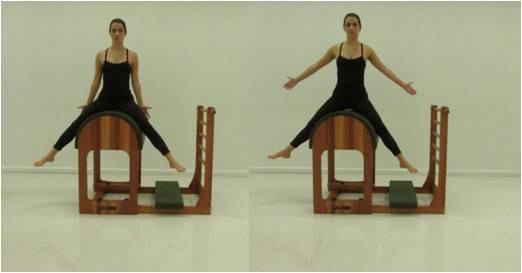  Objective: strengthen the adductor magnus, adductor longus, adductor minimus, pectineus and gracilis muscles. | *Teaser*  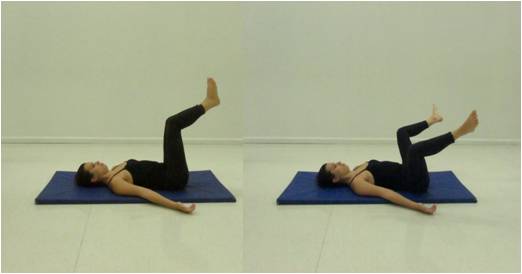  Objective: strengthen the adductor magnus, adductor longus, adductor minimus, pectineus and gracilis muscles. |
| *Stretches Front* (foot laterally)  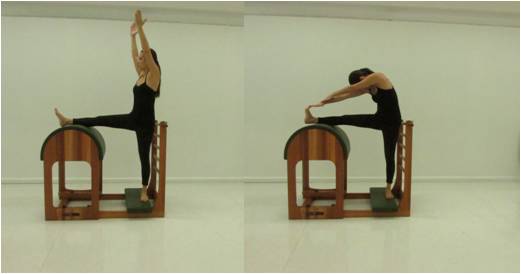  Objective: mobilize the vertebral column and lengthen the hamstring and triceps surae muscles. | *Stretches Fronts* (foot forward)    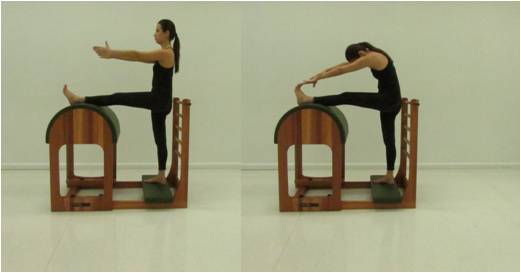  Objective: mobilize the vertebral column and lengthen the hamstring and triceps surae muscles. | *Leg Series*  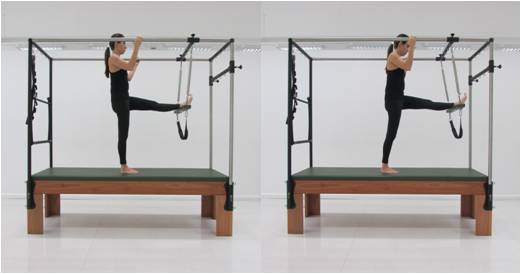  Objective: lengthen and strengthen the muscles from the posterior chain. |
| *Hamstring Stretch*    *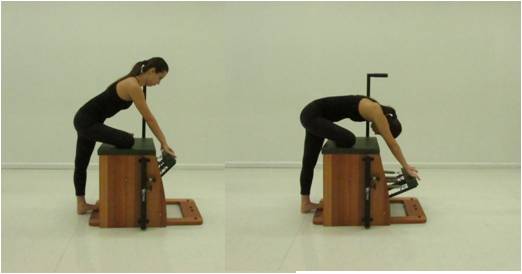*  Objective: mobilize the vertebral column and lengthen the muscles from the posterior chain. | *Stretches Front*  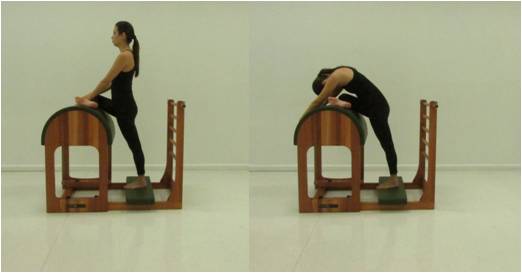  Objective: mobilize the vertebral column and lengthen the gluteus and hamstring muscles. | *Leg Stretch*  *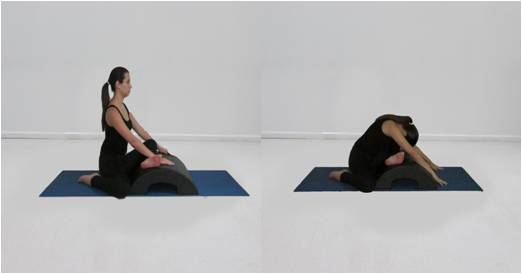*  Objective: mobilize the vertebral column and lengthen the gluteus, adductor magnus, adductor longus, adductor minimus, pectineus, gracilis and quadriceps femoris muscles. |

| **Exercises for the Upper Limbs (8)** | | |
| --- | --- | --- |
| **Basic** | **Intermediary** | **Advanced** |
| *Arms Biceps*  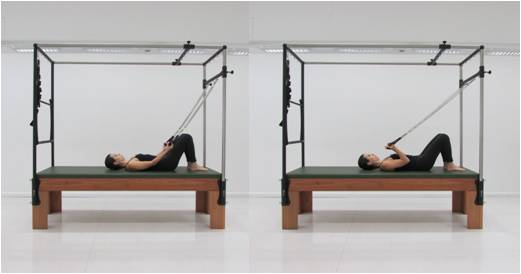  Objective: strengthen the biceps brachii and brachialis muscles. | *Arms Biceps Variant*  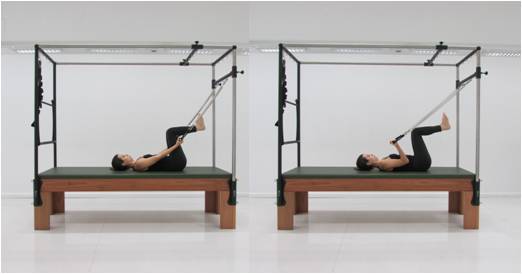  Objective: strengthen the biceps brachii and brachialis muscles. | *Arms Biceps Variant*  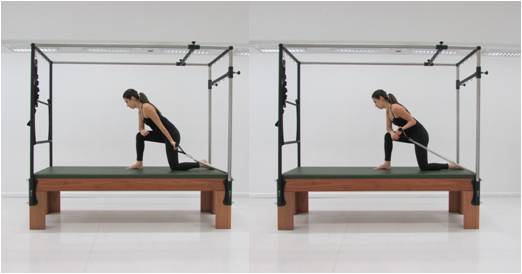  Objective: strengthen the biceps brachii, brachialis, rectus abdominal, iliopsoas and rectus femoris muscles. |
| *Arms Biceps*  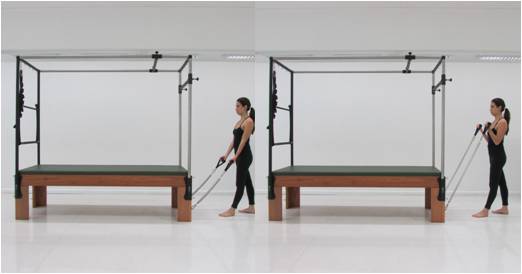  Objective: strengthen the biceps brachii, brachialis and brachioradialis muscles. | *Arms Biceps*  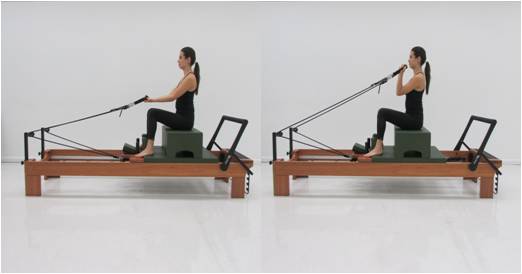  Objective: strengthen the biceps brachii, brachialis and brachioradialis muscles. | *Arms Biceps Variant*  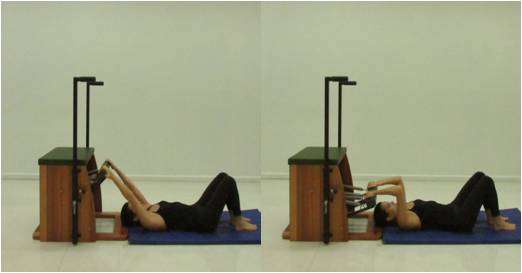  Objective: strengthen the biceps brachii and brachialis muscles. |
| *Arms Pull Up and Down*  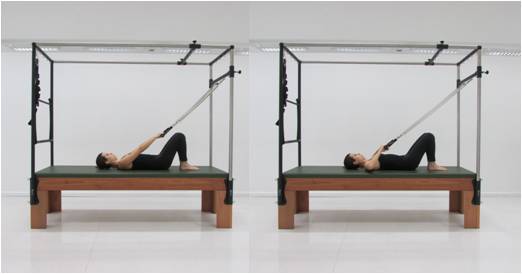  Objective: strengthen the deltoid and trapezius muscles. | *Arms Pulling*      Objective: strengthen the rhomboid, posterior fibers of deltoid, teres major, teres minor, infraspinatus, supraspinatus, and middle and inferior fibers of trapezius muscles. | *Arms Pulling Variant*    Objective: strengthen the rhomboid, posterior fibers of deltoid, teres minor, infraspinatus, supraspinatus and middle and inferior fibers of trapezius muscles. |
| *Arms Pull Up and Down Variant*    Objective: strengthen the pectoralis and latissimus dorsi muscles. | *Arms Pull Up and Down Variant*    Objective: strengthen the deltoid, latissimus dorsi, teres major, pectoralis major and coracobrachialis muscles. | *Long Box Pulling Straps*    Objective: strengthen the latissimus dorsi, teres major, pectoralis major and coracobrachialis muscles. |
| *Arms Pulling*    Objective: strengthen the rhomboid, latissimus dorsi, posterior fibers of deltoid, biceps brachii, middle and inferior fibers of trapezius and teres minor muscles. | *Arms Pulling*      Objective: strengthen the rhomboid, latissimus dorsi, posterior fibers of deltoid, teres minor and middle and inferior fibers of trapezius muscles. | *Arms Pulling*    Objective: strengthen the muscles romboids, latissimus dorsi, teres minor, posterior deltoid and middle and inferior fibers of trapezius. |
| *Arms Triceps*    Objective: strengthen the triceps brachii muscle. | *Arm Triceps*    Objective: strengthen the triceps brachii and anconeus muscles. | *Standing On Floor At Open End Boxe*      Objective: strengthen the triceps brachii muscle. |
| *Triceps Sit* (use a box)    Objective: strengthen the triceps brachii muscle. | *Triceps Sit On Foot Bar*      Objective: strengthen the triceps brachii and anconeus muscles. | *Triceps Front*      Objective: strengthen the triceps brachii, anconeus and pectoralis major muscles. |
| *Arms Pushing Variant*    Objective: strengthen the anterior and middle fibers of deltoid, triceps brachii and pectoralis muscles. | *Arms Pushing Variant*    Objective: strengthen the anterior and middle fibers of deltoid, triceps brachii and pectoralis muscles. | *Exercise On the Ball*    Objective: strengthen the pectoralis, anterior and middle fibers of deltoid, and triceps brachii muscles and perform balance training. |

| **Exercises for the Abdomen (5)** | | |
| --- | --- | --- |
| **Basic** | **Intermediary** | **Advanced** |
| *Teaser*    Objective: lengthen the adductor magnus, adductor longus, adductor minimus, pectineus and gracilis muscles and strengthen the rectus abdominis and obliquus externus muscles. | *The Hundred Variant*    Objective: lengthen the adductor magnus, adductor longus, adductor minimus, pectineus and gracilis muscles and strengthen the rectus abdominal, oblique, latissimus dorsi and teres major muscles. | *Exercise On the Ball*    Objective: strengthen the rectus abdominis and obliquus externus muscles. |
| *The Hundred* (pumping the arms)    Objective: strengthen the rectus femoris and obliquus externus muscles. | *The Hundred*    Objective: strengthen the rectus abdominis, obliquus externus, obliquus internus, latissimus dorsi, teres major and quadriceps femoris muscles. | *The Hundred Variant*    Objective: strengthen the rectus abdominis, obliquus externus, obliquus internus, latissimus dorsi, teres major and quadriceps femoris muscles. |
| *The Hundred Variant*    Objective: lengthen the rectus abdominis, obliquus internus and externus, iliopsoas, pectoralis and triceps brachii muscles. | *Exercise On the Ball*    Objective: perform balance training and globally strengthen the muscles (pectoralis, triceps brachii, rectus abdominis, obliquus externus, obliquus internus, iliopsoas, quadriceps and triceps surae). | *Exercise On the Ball*    Objective: perform balance training and globally strengthen the muscles (pectoralis, triceps brachii, rectus abdominis, obliquus externus, obliquus internus, iliopsoas, quadriceps and triceps surae). |
| *Sit-Up*      Objective: lengthen the muscles from the anterior chain and strengthen the rectus abdominis and obliquus externus muscles. | *Sit-Up* *Variant*    Objective: lengthen the muscles from the anterior chain and strengthen the rectus abdominis and obliquus externus muscles. | *Sit-Up* *Variant*    Objective: lengthen the muscles from the anterior chain and strengthen the rectus abdominis, obliquus externus, quadriceps femoris and iliopsoas muscles. |
| *Sit-Up*      Objective: mobilize the vertebral column and strengthen the rectus abdominis muscle. | *Sit-Up Variant*    Objective: mobilize the vertebral column and strengthen the rectus abdominis and quadriceps femoris muscles. | *Sit-Up Variant*    Objective: mobilize the vertebral column and strengthen the rectus abdominis, iliopsoas and quadriceps femoris muscles. |
